# Peach Water Relations, Gas Exchange, Growth and Shoot Mortality under Water Deficit in Semi-Arid Weather Conditions

**DOI:** 10.1371/journal.pone.0120246

**Published:** 2015-04-01

**Authors:** Mitra Rahmati, Gholam Hossein Davarynejad, Michel Génard, Mohammad Bannayan, Majid Azizi, Gilles Vercambre

**Affiliations:** 1 INRA, UR1115 Plantes et Systèmes de culture Horticoles, Domaine St Paul, Site Agroparc, Avignon, France; 2 Ferdowsi University of Mashhad, Faculty of Agriculture, Mashhad, Iran; South China Agricultural University, CHINA

## Abstract

In this study the sensitivity of peach tree (*Prunus persica* L.) to three water stress levels from mid-pit hardening until harvest was assessed. Seasonal patterns of shoot and fruit growth, gas exchange (leaf photosynthesis, stomatal conductance and transpiration) as well as carbon (C) storage/mobilization were evaluated in relation to plant water status. A simple C balance model was also developed to investigate sink-source relationship in relation to plant water status at the tree level. The C source was estimated through the leaf area dynamics and leaf photosynthesis rate along the season. The C sink was estimated for maintenance respiration and growth of shoots and fruits. Water stress significantly reduced gas exchange, and fruit, and shoot growth, but increased fruit dry matter concentration. Growth was more affected by water deficit than photosynthesis, and shoot growth was more sensitive to water deficit than fruit growth. Reduction of shoot growth was associated with a decrease of shoot elongation, emergence, and high shoot mortality. Water scarcity affected tree C assimilation due to two interacting factors: (i) reduction in leaf photosynthesis (-23% and -50% under moderate (MS) and severe (SS) water stress compared to low (LS) stress during growth season) and (ii) reduction in total leaf area (-57% and -79% under MS and SS compared to LS at harvest). Our field data analysis suggested a Ψ_stem_ threshold of -1.5 MPa below which daily net C gain became negative, i.e. C assimilation became lower than C needed for respiration and growth. Negative C balance under MS and SS associated with decline of trunk carbohydrate reserves – may have led to drought-induced vegetative mortality.

## Introduction

Water scarcity impacts various plant physiological processes and functions. These effects can be observed at various plant scales from the organs up to the whole plant. Water deficit affects shoot growth as well as fruit development [1]. Vegetative growth is highly sensitive to water deficit and can be reduced by drought—even more than fruit growth [1,2,3]. On the contrary, several studies have shown that root growth is less sensitive to water deficit than shoot and fruit growth [4]. In addition to growth reduction, water deficit increases the concentration of pulp soluble solids and dry mass content of fruits, through the reduction of water accumulation [5]. Two hypotheses have been suggested dealing with tree growth reduction under drought conditions [6]. One is associated with a reduction in carbon (C) acquisition. Responses such as transpiration reduction and photosynthetic limitation associated with stomatal closure have been documented for many trees [7]. The second hypothesis is associated with the hydraulic constraints limiting the cell expansion through a decrease in cell turgor [8]. When water absorption through the roots fails to keep up with transpiration, then loss of relative water content, decline of water potential and loss of turgor occur. The initial response of plants to water stress is growth reduction, even before any decrease in assimilation [9].

Water stress effects on plant phenology, growth and photosynthesis capacity could lead to a large effect on the C balance at the tree level. Under water stress conditions, tree C supply by photosynthesis and C demand by respiration and growth are not necessarily affected to the same extent [6]. Thus tree C reserves, key resource for bud-break to leaf area development, could be partially or totally depleted and led to questioning long-term tree’s growth, durability and survival [10]. Unlike forest trees, functioning and C economy of fruit trees under drought and high temperature conditions have rarely been studied. Both drought and high temperature can disrupt life processes and almost lead the tree to drought-related mortality [11,12].

In this research, we conducted a water stress experiment on a late-maturing peach (*Prunus persica* L.) under Iranian semi-arid climate conditions—low rainfall, high temperature and vapor pressure deficit and a maximum daily potential evapotranspiration as high as 15 mm. In this study, an insight into the sensitivity of various processes/organs to water deficit is provided with specific focus on (i) water relations, (ii) gas exchange, (iii) vegetative growth and mortality, (iv) fruit development, and (v) daily tree C balance and C gain. We hypothesized that the balance between C demand and C supply changed as drought intensified, resulting in possible C resource limitation. That assumption has been quantified by the daily C balance approach and discussed in the light of our experimental findings. A relationship between tree water status and tree C balance was also established, to indicate the progressive imbalance between C assimilation and C demand. This relationship can be extremely useful in irrigation management for peach orchards.

## Materials and Methods

### Study site and plant material

**Fig 1:**
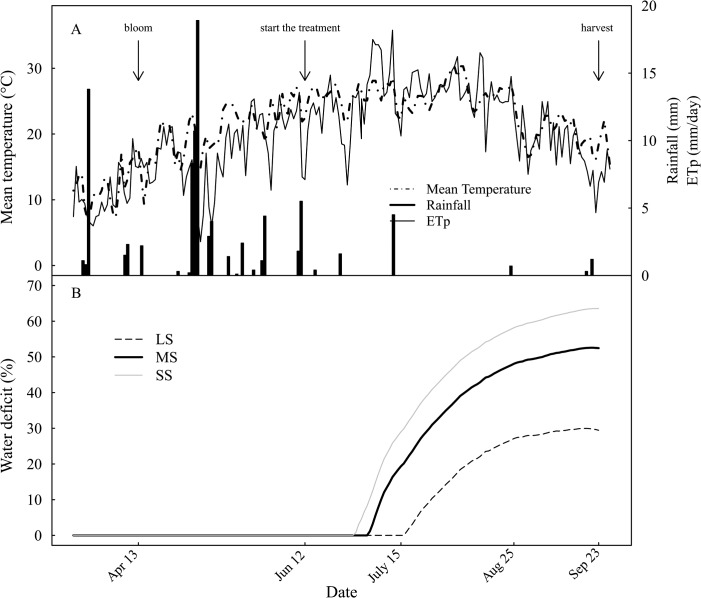
Diurnal courses of environmental conditions and water deficit intensity. (A) Air temperature, rainfall and reference evapotranspiration, ETp at the Golmakan meteorological station, Iran; (B) water deficit for different irrigation treatments during the 2011 growing season

This study was conducted during 2011, in Shahdiran commercial orchard, Golmakan, Iran (36° 29´ N, 59° 17´ E, around 1176 m above sea level). The orchard soil is sandy loam (64.0% sand, 30.0% silt) with pH 7.51 and 2.5 m soil depth. The average annual rainfall is about 212 mm. The maximum daily vapor pressure deficit was about 5.8 kPa and the maximum temperature ranged between 23°C and 38°C, at solar noon over the study period. Rainfall and reference evapotranspiration (ETp) were monitored near the orchard at Golmakan’s meteorological station (Fig 1A). The commercial orchard belongs to Shahdiran Company which has an agreement for research cooperation with Ferdowsi University of Mashhad and gave us permission to conduct the study on this site. Therefore, no specific permissions were required for these locations. The field studies did not involve endangered or protected species.

The vigorous growing eight-year-old ‘Elberta’ peach trees grafted on G.H.Hale seedling rootstocks were selected. The trees were spaced 4 × 5 m apart. Trees were managed according to commercial practices for fertilization, pest and weed control and hand thinning was done before employing any treatment, leading to a crop load of about 280 fruits per tree. Irrigation treatments were arranged in randomized complete block design with four replicate blocks per treatment. Each treatment plot consisted of 3 sub-blocks, each containing 3 trees. The measurements (plant water status, growth and composition, and gas exchange) were made on the central trees (4 trees per treatment) and the other trees were considered as guard trees. Moreover, root excavations were carried out, indicating that roots were mainly located near the dripper-line (50 cm from the rank) and that no root was crossing the inter-rank, due to severe drought and very low precipitation (data not shown).

Irrigation was carried out according to conventional irrigation applied in commercial orchards using a drip irrigation system with two lateral pipes per tree row and 6 emitters per plant. Accordingly, it was scheduled at 5 days per week for 3 hours each day. Each emitter delivered 8 L h^-1^ (low stress, LS—in other words, conventional irrigation), 4 L h^-1^ (moderate stress, MS) and 2 L h^-1^ (severe stress, SS) for the lowest-to-highest drought stress, respectively. Crop evapotranspiration (ET_c_) was calculated by multiplying ET_p_ by the crop coefficient (*K*_c_), in which the latter depends upon crop growth stages [13]. The soil water balance was estimated at full bloom depending upon the soil texture, soil water content and rooting depth [13]. Soil stored water and supplied water by precipitation and irrigation under different treatments compared to cumulative ETc during the growing season were used to estimate the water deficit (Fig 1B). Accordingly, the trees grown under conventional irrigation (LS) were subjected to no stress until mid-July and reached a water deficit of 30% at harvest (Fig 1B; Table 1), in other words the water supply was 30% less than cumulative ETc. The water deficit rose to 53% and 64% at harvest for MS to SS modalities, respectively, and was imposed 2–3 weeks earlier than that of LS modality.

**Table 1:** Monthly water balance components: crop evapotranspiration (ETc), Sum of rainfall, irrigation and soil supply for different irrigation treatments in the 2011 growing season.

		**Sum of rainfall, irrigation and soil supply (mm)**		
**Month**	**ETc (mm)**	**Low stress**	**Moderate stress**	**Severe stress**
Apr	6	94	94	94
May	80	306	306	306
June	254	469	422	397
July	604	632	507	442
Aug	950	790	588	482
Sep	1154	909	649	514

### Tree water status

Leaf water potential were measured at predawn (on August 13, 21 and 25 and September 8) and midday (June 23 and 27, July 15 and 24, August 13, 21 and 25 and September 8) using a pressure chamber (ELE, U.K.) on 3 fully expanded mature leaves—exposed to direct solar radiation—per tree (i.e. 12 per treatment). Early in the morning on August 13 and 21 and September 8, leaves were enclosed in plastic bags covered with aluminum foil for the measurements of stem water potential.

### Gas exchange measurements

Under clear-sky conditions, leaf assimilation (on June 23, July 1, 15 and 24, August 1, 13 and 25 and September 8 and 23) and transpiration (on June 23, July 1, 15 and 24 and August 1 and 13) were determined using a portable gas exchange system, LCA-4 ADC (Analytical Development Company, Hoddeson, England). Stomatal conductance and leaf temperature were computed on June 23 and 26, July 1, 15 and 24, August 1, 13, 21 and 25 and September 8 and 23 using Decagon SC-1 Leaf Porometer (Pullman, Washington). Measurements were taken on three fully-exposed upper-canopy leaves per tree, between 10 am and solar noon.

### Shoot and fruit growth measurements

A sample of 10 fruit bearing shoots (FBS) per tree that represented about 12% of the total number of FBS was selected from within the tree crown for measurements of leafy shoots and fruits growth. On each FBS, the number of leafy shoots and fruits were recorded, and the length of leafy shoots and the cheek diameter and height of fruits were measured every week from blooming stage to harvest. Shoot length was converted to dry mass based on an allometric relationship derived from data collected between 2011 and 2012. During the growing season up to harvest (6 dates), three fruits per tree (i.e. 12 per treatment) were sampled in order to determine the fresh mass, dry mass (oven dried at 70°C) and dry matter content.

Fruit relative growth rate (RGR) was calculated as:

RGR = (1/v)(dv/dt) (1)

where *v* and *t* represent fruit volume and time_,_ respectively. The peach fruit volume was calculated from mean fruit diameter assuming that the fruit was sphere.

At harvest, all leaves on a scaffold were collected and weighed to estimate total leaf fresh weight. Leaf area was measured with an area meter (LI-3100C model) for five samples of 20 g of leaves per treatment. Samples were then oven-dried at 60°C for at least two days, and their dry weights were determined, in order to assess specific leaf area (SLA, cm^2^ g^-1^), and leaf dry matter content (LDMC, g 100g^-1^). Scaffold leaf area was estimated by multiplying total leaf fresh weight per scaffold by leaf dry matter content (LDMC) and by specific leaf area (SLA). An allometric relationship between shoot length and leaf area were estimated. Leaf thickness (LT) was calculated based on SLA and LDMC (cm) at one unit of the leaf fresh density [14]:

$$LT\;=\;{\left(SLA\times LDMC\right)}^{-1}$$ (2)

### Trunk carbohydrate measurements

At harvest, three samples of trunk outer bark tissue (down to the wood) per tree (i.e. 12 per treatment) were collected. All trunk samples were immediately placed in liquid nitrogen, stored at -20°C, freeze-dried and grounded in liquid nitrogen. Glucose, fructose, sucrose, sorbitol and starch were extracted, and enzymatic analyze were carried out using a micro-plate reader [15].

### Tree carbon balance

A simplified source/sink approach was used to estimate tree carbon (C) balance. C source at the tree level was calculated using the measured leaf area dynamics and leaf photosynthesis rate during the season. The C source was scaled up by multiplying sunlit leaf photosynthesis rate by a reducing factor—relating to light attenuation within the canopy—previously observed in peach trees [16]. Based on the shoot and fruit growth measurements during growing season, the carbohydrate need for maintenance respiration and growth was estimated. The maintenance respiration is proportional to the dry mass, a maintenance respiration rate and a Q10 law to incorporate the effect of temperature [17]. The growth cost (respiration and dry biomass accumulation) was estimated using a growth efficiency parameter for leafy shoots [18] and for fruits [19]. The C concentration was 42% of the dry mass for the vegetative part [20], and 47.5% for the fruit [19]. Daily net C gain was computed as the daily difference between C source and C maintenance, and growth costs [21].

### Data analysis

All statistical analyses were made using R 2.15.0 software (R Development Core team, 2010). The comparison of mean values for the three irrigation levels was made by one-way ANOVA, followed by the Fisher’s least significant difference (LSD) test at the significant level of 0.05.

## Results

### Tree water status

**Fig 2:**
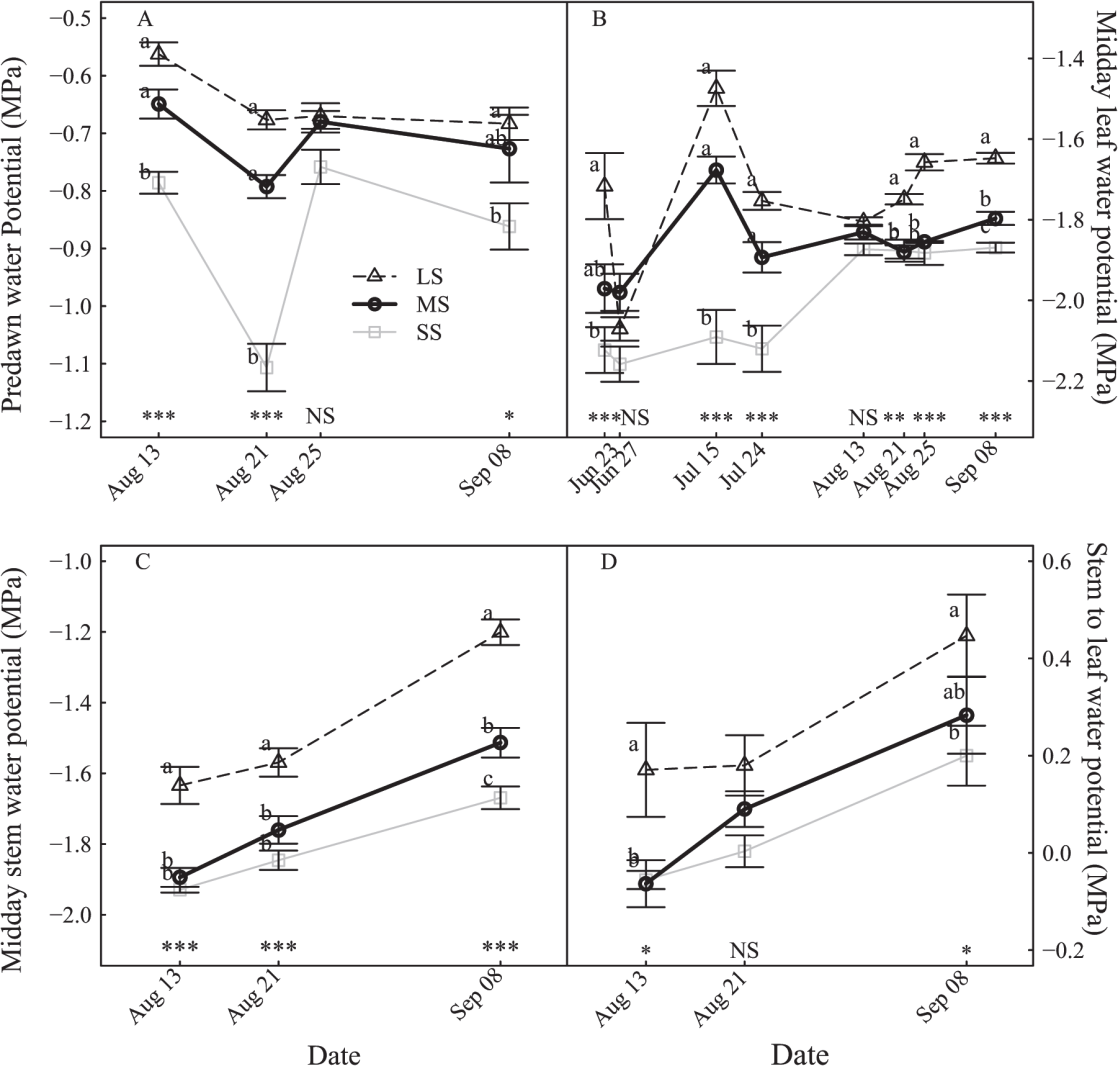
Evolution of tree water status under different irrigation levels. (A) Predawn water potential, midday (B) leaf and (C) stem water potential and (D) stem to leaf water potential difference. Abbreviations: LS = low stress, MS = moderate stress and SS = severe stress treatment. Data are mean values of 12 replicates ± standard error. Differences between the irrigation treatments were either significant at P < 0.10 (*), P < 0.05 (**) or P < 0.01 (***) or non-significant (NS) on each sampling date.

The different irrigation modalities significantly affected the plant water status. The SS treatment reached lower predawn water potential values than the LS and MS treatments (Fig 2A), except on the 25th of August which coincided with rain occurrence. From the 23rd of June onwards, i.e. less than 11 days after the treatment started, the SS midday leaf water potential was significantly lower than that of LS (Fig 2B). Significant difference between the LS and the MS treatment (Fig 2B) was observed 70 days after the treatment onset. The midday stem water potential significantly differed between the irrigation treatments at most of the measuring dates (Fig 2C). The stem to leaf water potential difference greatly decreased and it reached almost zero under MS and SS treatments (Fig 2D).

### Leaf gas exchanges

**Fig 3:**
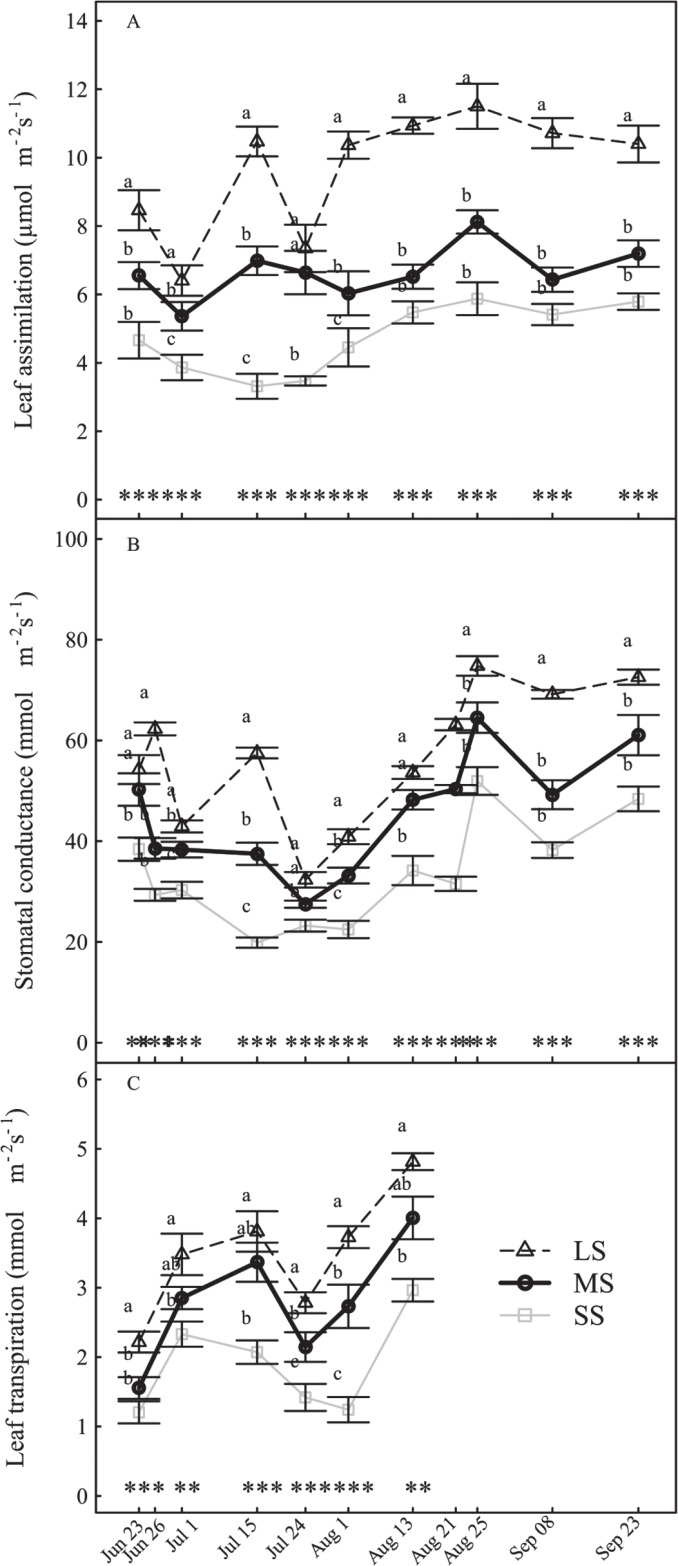
Evolution of the leaf gas exchange under different irrigation levels. (A) Assimilation, (B) Stomatal conductance and (C) Transpiration. Data are mean values of 20 replicates ± standard error. Labels and statistics are described in Fig 2.

The leaf gas exchanges were significantly affected by the irrigation treatments. Indeed, the photosynthesis, stomatal conductance and transpiration were reduced by up to 25% for trees under MS compared to trees under LS treatment, during the season (Fig 3A, 3B and 3C). The reduction in gas exchange was up to about 50% for trees under SS compared to the trees under LS treatment (Fig 3A, 3B and 3C).

### Gas exchange versus midday leaf water potential

**Fig 4:**
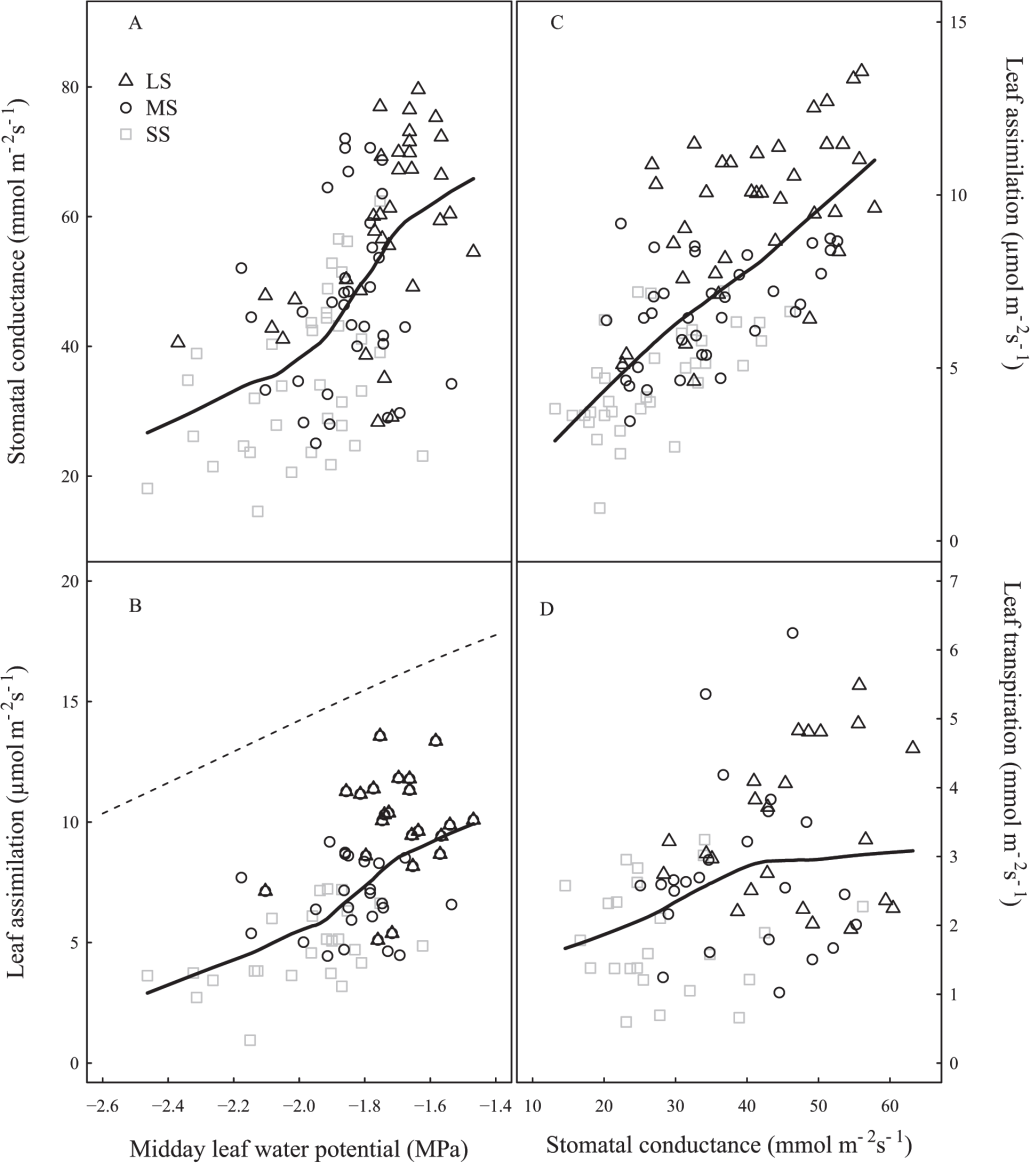
Relations between tree water status and leaf gas exchanges. (A) Relations between stomatal conductance and midday leaf water potential, (B) Relations between leaf assimilation and midday leaf water potential (C) Relations between leaf assimilation and stomatal conductance and (D) Relations between leaf transpiration and stomatal conductance. Each point represents the average of 3 measurements on a tree. Labels and statistics are described in Fig 2. In B, the dotted line indicates the predicted values of maximum photosynthesis from Ben Mimoun et al. equation [22]. All curves are smoothed fits of the data points using the ‘lowess’ method (R Development Core team, 2010).

Stomatal conductance and leaf assimilation were plotted as a function of midday leaf water potential (Ψ_midday_) (Fig 4A and 4B). Stomatal conductance and leaf assimilation reduced more than 50% when Ψ_midday_ decreased from -1.4 to -2 MPa. However, additional decrease in Ψ_midday_ (values lower than -2 MPa) only led to a slight decrease in stomatal conductance and leaf assimilation. Fig 4B also shows that leaf assimilation values of LS trees were always lower than maximum photosynthesis values previously calculated for peach trees [22]. Leaf assimilation is in association with stomatal conductance (Fig 4C), and maximum rate of leaf assimilation was achieved at the highest stomatal conductance. However, for a given stomatal conductance, leaf assimilation of MS and SS trees was lower than that of LS trees (Fig 4C). Although there was a tendency for leaf transpiration to increase with increasing stomatal conductance, the transpiration rate reached a plateau against stomatal conductance greater than 40 mmol m^-2^ s^-1^ (Fig 4D).

### Vegetative growth

**Fig 5:**
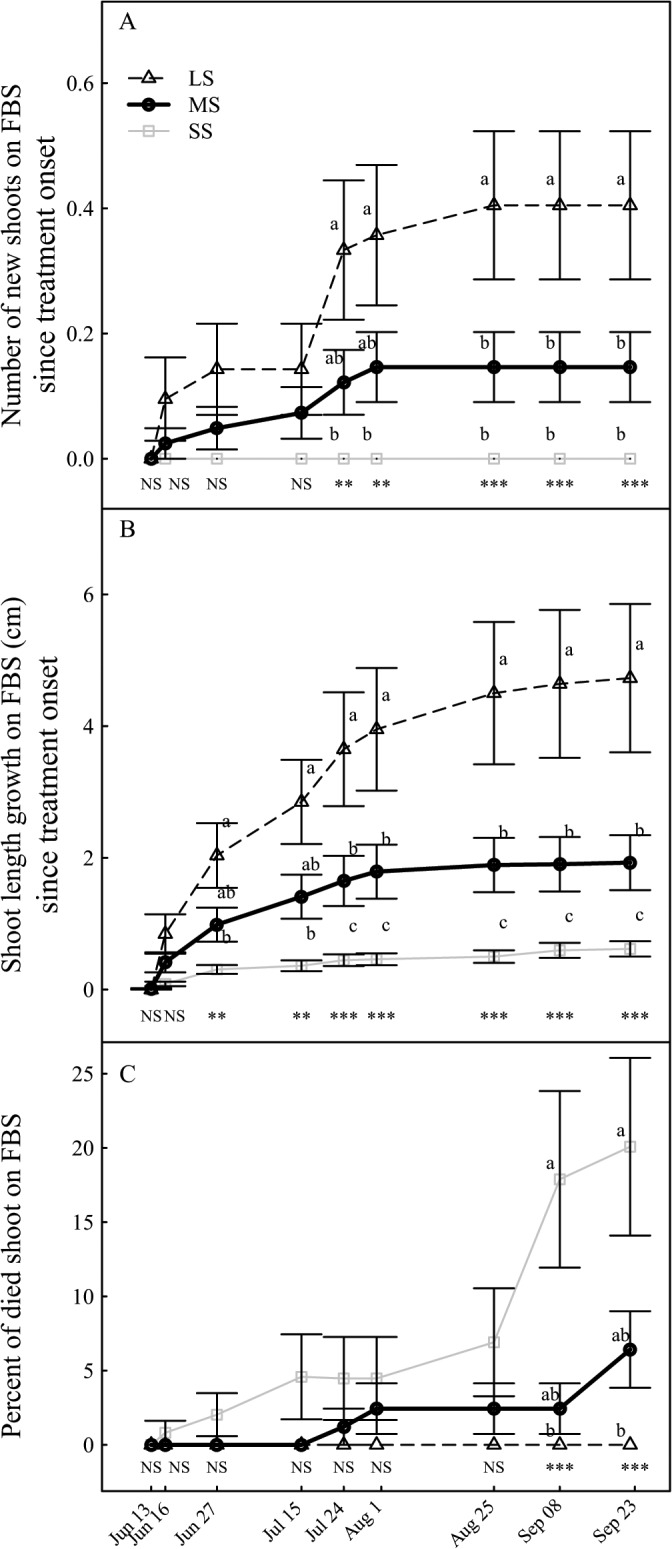
Evolution of the vegetative growth under different irrigation levels. (A) Emergence of shoots on the FBS since treatment onset. (B) Shoot length growth since treatment onset. (C) Shoot mortality. Data are mean values of 40 replicates ± standard error. Labels and statistics are described in Fig 2.

**Fig 6:**
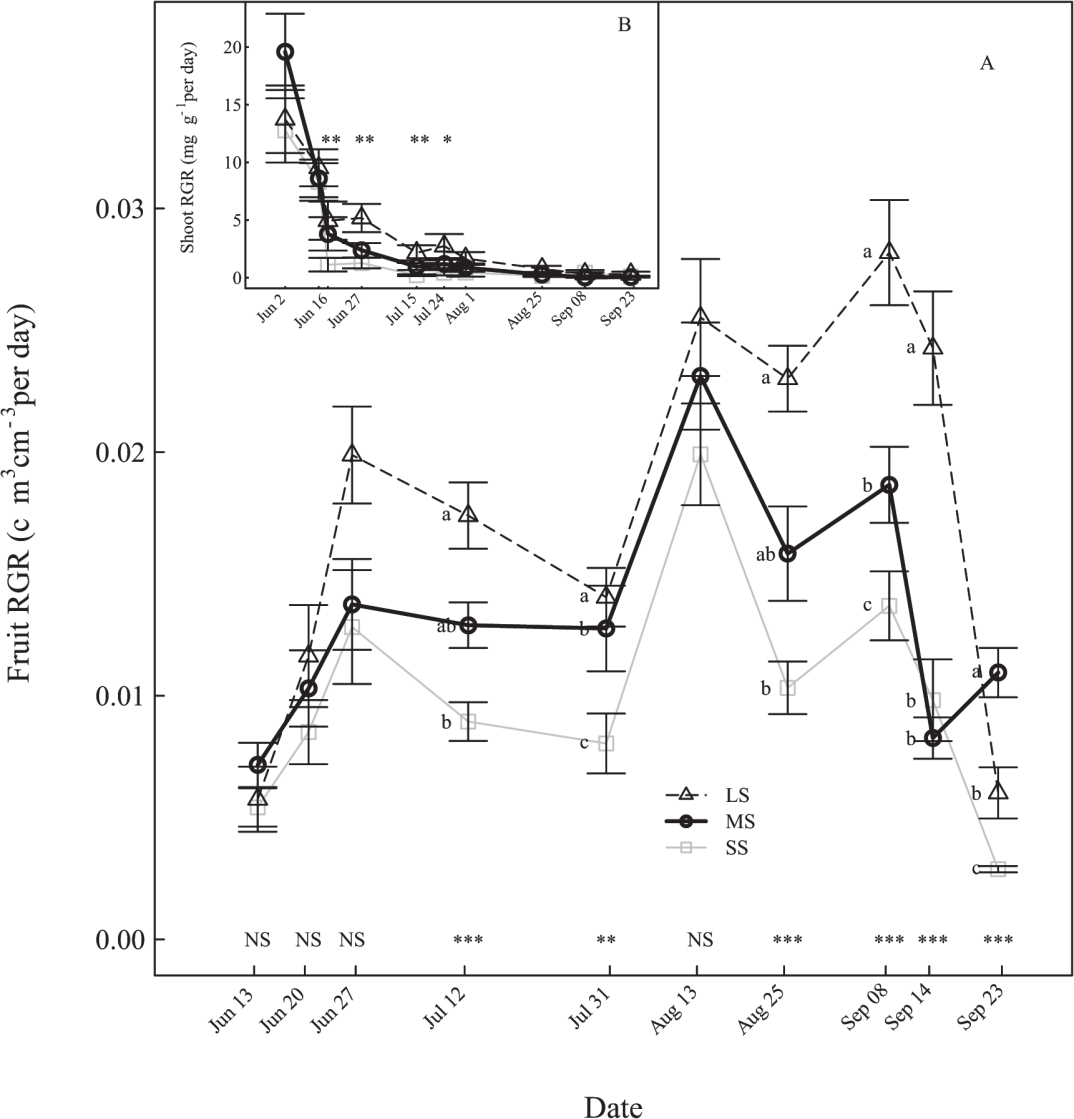
Dynamic changes of relative growth rate (RGR) under different irrigation levels. (A) Fruit RGR. (B) Shoot RGR. Data shows mean values ± standard error. Labels and statistics are described in Fig 2.

For LS trees, the mean shoot length growth duration was about 100 days since the vegetative bud-break, whereas it was shortened by 24 days and 60 days for the MS and SS compared to LS, respectively. Shoot length (Fig 5B) of LS trees rapidly increased from the end of fruit set and then reached a plateau. Significant reduction in shoot length growth occurred from 42 days (Jul 24th) and 15 days (Jun 27th) after the treatment onset for the MS and the SS trees compared to LS trees, respectively. Furthermore, shoot relative growth rate (RGR) was significantly declined during the main growth stage (Fig 6B). At fruit harvest, the mean shoot length for MS and SS trees was, respectively, 44% and 72% shorter than that of LS trees.

The seasonal pattern of leafy shoot emergence of LS trees exhibited a two-step increase followed by the complete halt of leafy shoot emergence (Fig 5A). The irrigation levels had significant effects on the emergence of leafy shoots on the FBS. Two and a half month after the treatment onset, the total number of new leafy shoots on the FBS for MS trees only represented 36% of that of the LS trees. For SS trees, no shoot emerged on the FBS throughout the season.

Shoot mortality was monitored due to water stress (Fig 5C). Up to about 7% and 19% of previously emerged leafy shoots were died three months after treatment onset for MS and SS trees, respectively (Fig 5C).

Specific leaf area (Table 2) declined by 10% for MS trees and up to 31% for SS trees compared to LS trees. The leaf dry matter content (LDMC) of SS trees was about 30% greater than that of LS trees, but no significant increase in LDMC was observed for MS trees. At harvest, the leaves of MS and SS trees were respectively, 7% and 10% thicker than those of LS trees (Table 2). Such an impact of water deficit on shoot emergence, growth and mortality led to a large reduction in the tree leaf area (LA). The LA of MS and SS trees at harvest were only 43% and down to 19% of that of LS trees (Table 2) respectively. Leaves on axillary shoots (sylleptic ramification) and watersprouts contributed to 54% of the total LA at harvest in LS trees but only to 23% in SS trees. Leaf dry weight (LDW) of MS and SS trees was only from 47% down to 27% of that of LS trees, respectively (Table 2). Such a larger decrease in LA compared to LDW under SS shows that an increase in drought intensity affected growth per unit surface severer than that per unit dry mass.

**Table 2:** Leaf dry matter content (LDMC), specific leaf area (SLA), leaf thickness (LT) and leaf dry weight (LDW) and area per peach tree (LA) at harvest for different irrigation treatments.

**Treatment**	**LDMC (g 100g** ^-1^ **)**	**SLA (cm** ^2^ **g** ^-1^ **)**	**LT (×10** ^–2^ **cm)**	**LDW per tree (g)**	**LA per tree (m** ^2^ **)**	**Proportion of LA on axillary shoots and water sprouts (%)**
Low stress	26.78 ± 2.28 b	157.57 ± 1.12 a	2.57 ± 0.31 b	1880 ± 152 a	29.5 ± 2.4 a	54 ± 5 a
Moderate stress	27.65 ± 1.64 b	142.83 ± 0.78 ab	2.68 ± 0.18 b	888 ± 73 b	12.8 ± 1.0 b	32 ± 9 ab
Severe stress	34.91 ± 2.13 a	108.92 ± 0.98 b	2.76 ± 0.17 a	500 ± 85 c	5.6 ± 0.9 c	23 ± 8 b

Data shows mean values of twelve replicates ± standard error. In each column, values with the same letter are not significantly different.

### Fruit growth

**Fig 7:**
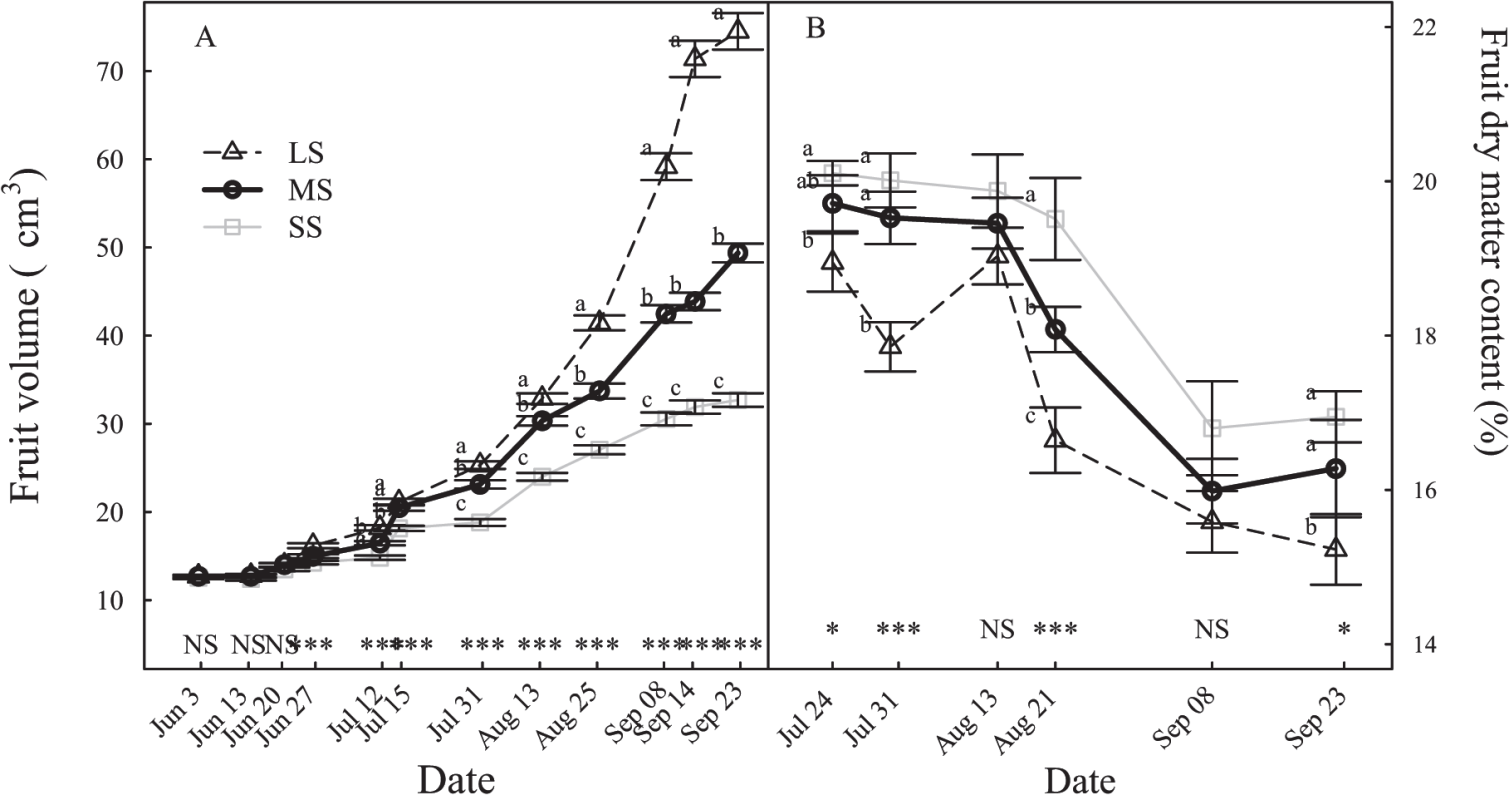
Evolution of the fruit growth under different irrigation levels. (A) Fruit volume. (B) Fruit dry matter content. Data shows mean values ± standard error. Labels and statistics are described in Fig 2.

The reduction of fruit growth started 15 days after the treatment onset, which at harvest resulted in reduction of fruit volume by 34% and 56% on MS to SS trees compared to that of LS trees, respectively (Fig 7A). LS fruit dry matter content (DMC) decreased throughout fruit growth from 19% to 15% of fresh weight (Fig 7B). The fruit DMC of SS trees was significantly increased compared to LS trees on most sampling dates. At harvest, fruit dry matter content increased by 7% for MS and 11% for SS fruits compared to that of LS fruits (Fig 7B). In contrast to the seasonal pattern of shoot RGR which showed a gradual decrease throughout the season (Fig 6B), fruit RGR showed a typical pattern with two large growth periods, suspended during the pit hardening stage (Fig 6A). The Fruit RGR under SS was significantly lower than that of LS from one month after the treatment onset until harvest.

### Tree carbon balance and trunk carbohydrate contents

**Fig 8:**
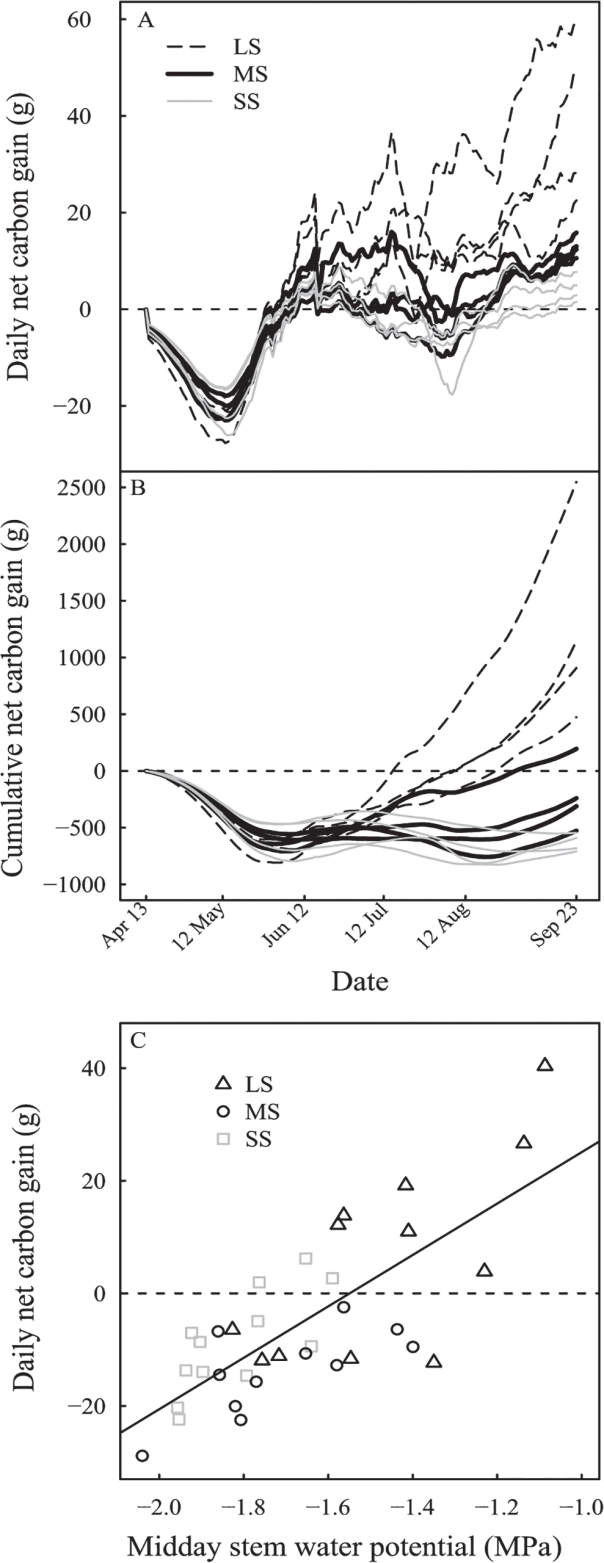
Evolution of net carbon gain under different irrigation levels. (A) Daily net carbon gain. (B) Cumulative net carbon gain. (C) Relationship between daily C gain and midday stem water potential. Labels are described in Fig 2.

Fig 8A and 8B shows the dynamic changes of net carbon (C) gain throughout the season for trees grown under the different irrigation levels. The daily net C gain is always negative until the leaf area is established, coinciding with mid-pit hardening, which means that previously stored C is used for growth. Afterward it greatly increased from zero and reached about an average of 19 g day^-1^ for LS trees, while it did not increase much above zero for SS trees (Fig 8A). LS trees accumulated excess C from about two months before harvest. Cumulative C balance was greatly affected by the water stress intensity, and the C balance was always negative under MS and LS treatments (Fig 8B). Daily net C gain decreased linearly with decreasing midday stem water potential. The daily C balance is null for a threshold of -1.5 MPa (Fig 8C). Below this threshold, the daily C balance is negative. Such negative C balance indicates that carbohydrates were retrieved from storage organs in order to sustain the C demand for the organs’ growth and respiration. Compared to LS trees, MS and SS trees starch concentration decreased by 34% and 52% respectively. Among the non-structural carbohydrates (NSC) in the trunk (Table 3), sorbitol was not significantly affected by the irrigation treatment, whereas glucose and fructose decreased by about 19% and 12%, for SS compared to LS, respectively. The decrease in both starch and total soluble sugars led to a significant decrease in total NSC concentrations.

**Table 3:** Non-structural carbohydrate (NSC) concentrations of peach trunk after harvest for different irrigation treatments.

	**Concentration (g 100g** ^-1^ **of dry weight)**		
**Carbohydrate**	**Low stress**	**Moderate stress**	**Severe stress**
Sorbitol	1.39 ± 0.05 a	1.38 ± 0.02 a	1.40 ± 0.04 a
Glucose	3.02 ± 0.13 a	2.69 ± 0.12 ab	2.42 ± 0.12 b
Fructose	3.24 ± 0.09 a	3.00 ± 0.06 ab	2.83 ± 0.05 b
Total sugars	7.70 ± 0.21 a	7.30 ± 0.16 ab	6.75 ± 0.15 b
Starch	1.53 ± 0.20 a	1.01 ± 0.12 b	0.74 ± 0.08 b
Total NSC	9.26 ± 0.28 a	8.29 ± 0.27 b	7.49 ± 0.18 c

Data are mean values of twelve replicates ± standard error. Values followed by the same letter within a row are not significantly different.

## Discussion

In this study severe drought affected the plant water status through the reductions of stem, and leaf water potentials. These results are in agreement with Besset *et al*. [23]. Furthermore, the reduction of stem water potential was larger than the reduction of leaf water potential. This is consistent with other reports indicating that midday stem water potential is a better indicator of tree water status than midday leaf water potential [24,25]. The water deficit reduced the stem to leaf water potential difference. Since the movement of water from the stem to the leaf is driven by the stem to leaf water potential difference, the results of this study indicate that the water flow to the leaf was severely to totally blocked for SS trees. These observations are in agreement with severe reductions in leaf transpiration measurements as drought intensified.

Peach maximum leaf photosynthetic potential is around 20–22 μmol m^-2^ s^-1^ [22,26]. However, in our experiment, it did not increase above 12 μmol m^-2^ s^-1^ in LS trees during the season. Furthermore, stomatal conductance was low (less than 100 mmol m^-2^ s^-1^) compared to other studies [7]. Such low leaf assimilation and stomatal conductance could be due to the low leaf water potential measured in our study, even for the LS modality. Decreased leaf assimilation and transpiration associated with limited stomatal conductance have previously been reported for fruit trees under drought [5,27,28] and it has been concluded that the reduction in leaf assimilation during a drought is probably due to stomatal closure occurring when leaf water potential declines below a threshold value. However, greater leaf assimilation for LS trees than for SS trees for a given stomatal conductance suggests another limiting factor. In the field, heat stress often accompanies water deficit through the limitation of the transpiration rate and the increase in leaf temperature (data not shown). This increase in leaf temperature could lead to a decrease in the activation state of Rubisco, or impaired ATP synthesis, leading to photosynthesis limitation [29,30]. Furthermore, mesophyll conductance to CO_2_ is a key variable for the photosynthesis process, leading to possible limitation in the CO_2_ concentration to the chloroplast. Water deficit can induce a rapid reduction in mesophyll conductance [29,31] which leads to reduction of photosynthesis by 20%-50%. Mesophyll conductance could be linked to leaf structure [32,33]. Denser and thicker leaves with lower specific leaf area are likely to have lower mesophyll conductance [31,34]. An increase in leaf dry matter content and thickness and an associated decrease in the specific leaf area were observed in our study, as previously reported under drought condition [35,36]. However, as leaf assimilation was more strongly affected by water deficit than leaf transpiration, the decline in mesophyll conductance cannot be the only limiting factor to reduce leaf assimilation, as it should similarly affect water and CO_2_ diffusion within the mesophyll. Moreover, some studies have addressed the indirect effects of water deficit on the limitation of leaf assimilation. Since growth is more severely affected than photosynthesis [9], extra-assimilated carbon (C) is either stored as sugars or converted to starch for storage. The accumulation of NSC has an impact on photosynthetic capacity through feedback inhibition [8,37]. Therefore, with regards to the large growth decline under water stress in this experiment, the decrease in leaf assimilation could also be related to a feedback inhibition of accumulated NSC in the leaves. Therefore, in association between leaf photosynthesis and drought stress, leaf photosynthesis could be reduced in three ways: an increase in stomata and mesophyll resistance, a rise in leaf temperature, and a negative feedback of stored carbohydrates within leaves.

Water deficit greatly reduced the shoot emergence on FBS, and especially during the end of the fruit pit hardening. Indeed, SS trees showed no shoot emergence. Moreover, the leafy shoots grown under water stress were shorter. Growth reduction appeared quickly after the treatment onset under SS, and lasted until the end of the vegetative growth. Shorter duration of vegetative growth and a lower relative growth rate of the shoots were associated with drought. A shortened growth period and a slower radial growth rate for low soil water potential in woody plants were reported [38]. Our results as reduction of shoot emergence and growth, are in agreement with the finding of Hipps et al. [39] who showed that the shoot system architecture of young peach trees was significantly affected by water availability. This is directly attributed to the decrease of water potential, rather than to the constraints on C availability [8,38]. However, other studies indicated that under prolonged drought, maintenance respiration was maintained, while photosynthesis was severely reduced. This phenomenon probably resulted in the carbohydrates limitations and thereby a decrease in organs growth [6,40,41].

Water stress had a large impact on shoot mortality. The injury was visible as a yellowing or withering of the leaves, shriveling green fruits and drying of the wood. At the end of the vegetative growth, 20% of previously emerged leafy shoots on SS trees were dry. In contrast, water deficit imposed during the harvest period on almond (*Prunus dulcis*) trees, had no effect on spur mortality [42]. In this case, water stress occurred very late, long after completion of the vegetative growth cycle, and thus did not impact shoot mortality. McDowell [11] has reviewed the mechanisms of drought-induced mortality. Possible explanations rely either on (i) hydraulic failure or (ii) C starvation or a coupling of both processes [11,43]. Hydraulic failure is linked to xylem embolism, blocking the water flow within the xylem network [44] and thus the shoot water supply. When C supply from photosynthesis and mobilization of NSC is lower than C demand during respiration, it could lead to a large decrease in carbohydrate content which induces shoot mortality. It has been suggested that higher temperature associated with drought may result in a more rapid depletion of carbohydrate reserves associated with tissue mortality [45]. Similar results were obtained in this study. A vulnerability curve for the embolism and native characterization of the percent loss of conductivity could help to determine the origin of shoot mortality [12].

As drought intensified the reduction of leaf area (LA) was more than leaf dry weight (LDW) per tree. Such results indicate the higher sensitivity of vegetative growth per surface unit compared to growth per unit of dry mass. Such reduction of the LA has been already reported for almonds and peach seedlings under water stress [2,27]. The reduction in LA was primarily caused by a large reduction in shoot growth, but also by a decrease in shoot emergence and an increase in shoot mortality. Similarly, the fruit volume was more affected than dry mass. Thus fruit dry matter content (DMC) increased as drought intensified, which implies that water inflow to fruit was more restricted than carbohydrate import for SS trees. Similar results were reported by Cui et al. [5].

Fruit dry mass of SS trees declined by 55% at harvest, whereas the leaves dry mass decreased by 73%. Thus fruit growth is less sensitive to water deficit. The water stress appeared during the fruit pit-hardening stage which is known to have very low sensitivity to drought [46]. Meanwhile, vegetative growth including shoot/leaf emergence and growth was still in progress, leading to a larger effect on the vegetative growth. As drought intensified, the tree C acquisition declined more than respiratory C loss, leading to limited tree C gain. The decrease in tree C acquisition was partly associated with a decrease in leaf photosynthesis, but the primarily limiting factor was the leaf area reduction. Our study demonstrated that SS led to a large depletion of carbohydrates in the trunk, i.e. starch reserve decreasing by 52% and glucose and fructose by 19% and 12%. These trees had experienced extended periods of negative C balance. Interestingly, sorbitol content, the main form in which C is translocated through phloem in the peach, was not affected by the irrigation levels. The content of trunk NSC are consistent with those were obtained by Da Silva et al. [47]. The depletion of C reserves in the trunk suggests that the previously stored C has been remobilized to sustain the growth of the fruits, the shoots and the root system in response to SS. For LS trees, the C balance was temporarily negative early in the season until part of the leaf area was developed. In SS trees, the C balance is always negative, even later in the season, leading to remobilization of the stored carbohydrates to meet aboveground and root C demands [47]. Besides, the decrease in carbohydrate storage during the season may have dramatic consequences for the tree lifespan, since the stored carbohydrate will be used for maintenance during winter and for bud burst and shoot growth the following spring [10]. Long C deficit periods associated with a depletion of C reserves may be a possible cause of drought-related vegetative mortality [11,48]. Drought-induced mortality due to decrease in starch reserves in woody plants under drought stress has been reported. For example, loss of starch in the roots of Norway spruce tree [40] or in the wood at collar of beech tree [49] which was associated with subsequent root mortality and extensive defoliation, respectively. This study data analysis showed a threshold midday stem water potential of -1.5 MPa below which peach daily C balance was negative. It means that stem water potential should be maintained higher than—1.5 MPa to prevent decrease of net C deficit. Such a threshold may be valuable information in order to manage irrigation under conditions of severe water scarcity.

## Conclusions

Water availability is a crucial determinant of C assimilation and C allocation mainly through altering organ emergence and growth within the tree. Water deficit affected tree C acquisition primarily by reducing the leaf area, and subsequently through a reduction of the leaf photosynthesis rate. The reduction in leaf area was mainly caused by the reduction in shoot growth, but also by the prevention of shoot emergence, and the induction of shoot mortality. Under these conditions, a midday stem water potential below a threshold of -1.5 MPa resulted in negative daily C balance. Such negative C balance associated with a depletion of carbohydrate storage may result in drought-induced vegetative mortality. The results of this study also confirmed that the vegetative growth was more strongly affected by water deficit than fruit growth. It would be interesting to include these results into functional-structural models of tree growth which have been developed for the peach tree [50,51] to provide more details of the whole-tree functioning in relation to water deficit.
